# A Self-Organizing Algorithm for Modeling Protein Loops

**DOI:** 10.1371/journal.pcbi.1000478

**Published:** 2009-08-21

**Authors:** Pu Liu, Fangqiang Zhu, Dmitrii N. Rassokhin, Dimitris K. Agrafiotis

**Affiliations:** Johnson & Johnson Pharmaceutical Research and Development, Exton, Pennsylvania, United States of America; Fox Chase Cancer Center, United States of America

## Abstract

Protein loops, the flexible short segments connecting two stable secondary structural units in proteins, play a critical role in protein structure and function. Constructing chemically sensible conformations of protein loops that seamlessly bridge the gap between the anchor points without introducing any steric collisions remains an open challenge. A variety of algorithms have been developed to tackle the loop closure problem, ranging from inverse kinematics to knowledge-based approaches that utilize pre-existing fragments extracted from known protein structures. However, many of these approaches focus on the generation of conformations that mainly satisfy the fixed end point condition, leaving the steric constraints to be resolved in subsequent post-processing steps. In the present work, we describe a simple solution that simultaneously satisfies not only the end point and steric conditions, but also chirality and planarity constraints. Starting from random initial atomic coordinates, each individual conformation is generated independently by using a simple alternating scheme of pairwise distance adjustments of randomly chosen atoms, followed by fast geometric matching of the conformationally rigid components of the constituent amino acids. The method is conceptually simple, numerically stable and computationally efficient. Very importantly, additional constraints, such as those derived from NMR experiments, hydrogen bonds or salt bridges, can be incorporated into the algorithm in a straightforward and inexpensive way, making the method ideal for solving more complex multi-loop problems. The remarkable performance and robustness of the algorithm are demonstrated on a set of protein loops of length 4, 8, and 12 that have been used in previous studies.

## Author Summary

Protein loops play an important role in protein function, such as ligand binding, recognition, and allosteric regulation. However, due to their flexibility, it is notoriously difficult to determine their 3D structures using traditional experimental techniques. As a result, one can often find protein structures with missing loops in the Protein Data Bank. Their sequence variability also presents a particular challenge for homology modeling methods, which can only yield good overall structures given sufficient sequence identity and good experimental reference structures. Despite extensive research, the construction of protein loop 3D structures remains an open problem, since a sensible conformation should seamlessly bridge the anchor points without introducing steric clashes within the loop itself or between the loop and its surroundings environment. Here, we present a conceptually simple, mathematically straightforward, numerically robust and computationally efficient approach for building protein loop conformations that simultaneously satisfy end-point, steric, planar and chiral constraints. More importantly, additional constraints derived from experimental sources can be incorporated in a straightforward manner, allowing the processing of more complex structures involving multiple interlocking loops.

## Introduction

The characterization of protein loop structures and their motions is essential in understanding the function of proteins and the biological processes they mediate [1],[2]. However, due to their conformational flexibility, it is notoriously difficult to uniquely determine their structure via traditional experimental techniques such as X-ray scattering or nuclear magnetic resonance (NMR). As a result, structures with missing loops are not uncommon in the Protein Data Bank. The sequence and structure variability of protein loops also presents a major challenge in homology modeling. With moderate sequence identity and good quality experimental template structures, it is generally feasible to obtain the overall tertiary structure and some acceptable degree of detail for the loop in question. However, the errors could be significant in the loop regions where the sequences between the target and template protein differ significantly. In our view, the loop closure problem, namely the construction of a protein fragment that closes the gap between two fixed end points, remains unsolved. A satisfactory solution to this problem will not only benefit experimental structure determination and comparative modeling, but also be useful in *de novo* protein structure prediction and phase space sampling, as the importance of local moves without changing the rest of the system has been repeatedly demonstrated for chain molecules [3],[4].

A complete solution to the protein loop reconstruction problem usually involves two important components, the buildup of the loop structure and the selection of the most promising candidates through an appropriate scoring function. The current study addresses the former problem. A variety of algorithms has been developed to tackle the loop closure problem. Many methods construct protein loops by reusing representative loop blocks from a database of experimentally determined protein structures [5]–[18]. Naturally, these methods are highly dependent on the size and quality of the experimental data, and their performance has improved substantially with the rapid growth of PDB [14],[15]. More importantly, since the number of possible conformations increases exponentially with length, this approach is limited to relatively short loops. This is not a problem for *ab initio* methods which construct loops by either distorting existing structures or by relaxing distorted non-physical structures with molecular dynamics, simulated annealing, gradient minimization, random tweaking, discrete (φ,ψ) dihedral angle sampling, or self-consistent field optimization [19]–[27]. These algorithms often include energy calculations using classical force fields and implicit or explicit treatment of solvent effects, and therefore tend to be computationally expensive. Several groups have combined knowledge-based and sampling approaches, sometimes with considerable success [10], [28]–[34]. For example, through modeling the crystal environment, careful refinements, and extensive conformational sampling, PLOP [33] obtained an average prediction accuracy of 0.84 and 1.63 Å RMSD from the crystal structures for a series of 8- and 11-residue loops. The performance of PLOP was further improved by Zhu and coauthors through an improved sampling algorithm and a new energy model [35], and was successfully applied even to loops in inexact environments [36].

An alternative class of methods determine proper loop structures by identifying all possible solutions to a set of algebraic equations derived from distance geometry, as described in the pioneering work of Go and Sheraga [37] and many other analytical methods adopted from kinematic theory [31], [38]–[41]. In particular, Canutescu and Dunbrack introduced a very attractive approach known as cyclic coordinate descent (CCD), which can close loops of different lengths through iterative adjustment of dihedral angles [40]. This method has been incorporated into the well-known *de novo* protein design package Rosetta and demonstrated its strength in generating conformations for the loop regions [42],[43]. More recently, Coutsias and coauthors cast the determination of loop conformations of six torsions into a problem of finding the real roots of a 16^th^ degree single-variable polynomial, and demonstrated the efficiency and applicability to various loops [41]. A thorough review of loop closure algorithms is beyond the scope of this paper. For more information, the reader is referred to several recent articles [31]–[34],[44].

In computational modeling, a protein loop can be conveniently represented by a set of connected points in three-dimensional Cartesian space. A chemically sensible conformation must satisfy a set of geometric constraints derived from the loop's covalent structure. The connectivity and common covalent bond lengths and angles require that the distance *d_ij_* between any pair of atoms *i* and *j* falls between certain bounds, 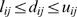. Non-bonded interactions introduce additional constraints, as do the planarity of conjugated systems and the chirality of stereocenters. These can be further supplemented with external constraints derived from experimental techniques such as 2D NMR and fluorescent resonance energy transfer (FRET). Taken together, these constraints greatly reduce the search space that needs to be sampled in order to identify the loop's accessible conformations. Distance geometry (DG) is a class of methods that aim specifically at generating conformations that satisfy such geometric constraints. DG attempts to minimize an error function that measures the violation of geometric constraints [45],[46]. DG methods involve four basic steps: 1) generating the interatomic distance bounds, 2) assigning a random value to each distance within the respective bounds, 3) converting the resulting distance matrix into a starting set of Cartesian coordinates, and 4) refining the coordinates by minimizing distance constraint violations. To ensure that reasonable conformations are generated, the original upper and lower bounds are usually refined using an iterative triangular smoothing procedure. Although this process improves the initial guess, the randomly chosen distances may still be inconsistent with a valid 3-dimensional geometry, necessitating expensive metrization schemes [47]–[49] or higher dimensional embeddings [46] prior to error refinement, or lengthy refinement procedures if random starting coordinates are used. Although DG methods can generate sensible starting geometries, these geometries are rather crude for most practical applications, and need to be further refined by some form of energy minimization. Since its first chemical applications in 1978 by Crippen and Havel [45], DG has been applied to a wide range of problems, including NMR structure determination, conformational analysis [48],[50], homology modeling [49],[51], and *ab initio* fold prediction [52].

Recently, a new self-organizing technique known as stochastic proximity embedding (SPE) has been developed as an extremely attractive alternative to conventional DG embedding procedures [53]. SPE starts from random initial atomic positions, and gradually refines them by repeatedly selecting an individual constraint at random, and updating the respective atomic coordinates towards satisfying that specific constraint. This procedure is performed repeatedly until a reasonable conformation is obtained. The method, which was originally developed for dimensionality reduction [54] and nonlinear manifold learning [55], is simple, fast and efficient, and can be applied to molecular topologies of arbitrary complexity (acyclic, cyclic, macrocyclic, bridged and caged systems alike). Because it avoids explicit evaluation of an error function that measures all possible interatomic distance bound violations in every refinement step, the method is extremely fast and scales linearly with the size of the molecule. SPE is significantly more effective in sampling the full range of conformational space compared to other conformational search methods [56], particularly when used in conjunction with conformational boosting [57], a heuristic for biasing the search towards more extended or compact geometries. Furthermore, SPE is insensitive to permuted input, a problem that plagues many systematic search algorithms [58].

Zhu and Agrafiotis subsequently proposed an improved variant of SPE referred to as self-organizing superimposition (SOS) that accelerates convergence by decomposing the molecule into rigid fragments and using pre-computed conformations for those fragments in order to enforce the desired geometry [59]. Starting from completely random initial coordinates, the SOS algorithm repeatedly superimposes the templates to adjust the positions of the atoms, thereby gradually refining the conformation of the molecule. Coupled with pair-wise atomic adjustments to resolve steric clashes, the method is able to generate conformations that satisfy all geometric constraints at a fraction of the time required by SPE. The approach is conceptually simple, mathematically straightforward, and numerically robust, and allows additional constraints to be readily incorporated. Since rigid fragments are pre-computed, planarity and chirality constraints are automatically satisfied after the template superimposition process, and local geometry is naturally restored. Furthermore, because each embedding starts from completely random initial atomic coordinates, each new conformation is independent of those generated in the previous runs, resulting in greater diversity and more effective sampling. As the algorithm only involves pairwise distance adjustments and superimposition of relatively small fragments, it is impressively efficient.

In this paper, we present the new variant of the SOS algorithm, which has been adapted from conformational sampling of small molecules and tailored to the protein loop closure problem. In the remaining sections, we provide a detailed description of the modified SOS algorithm and its implementation, and present comparative results for a set of protein loops of residue size 4, 8, and 12, which have been used in previous validation studies.

## Methods

The SOS algorithm involves two main phases: 1) an *initialization* phase, where the input molecule is decomposed into a set of rigid fragments, and the upper and lower inter-atomic distance bounds are determined; and 2) an *embedding* phase, where molecular conformations consistent with these distance bounds are generated through a series of alternating template fitting and pairwise distance adjustments.

### 

#### Initialization

The initialization process is applied once for each new molecule and involves three basic steps:

1. Decompose the target molecule into overlapping fragments [59] and retrieve the ideal conformational template *T_i_* for each fragment *F_i_* from a library of pre-computed templates (since there is a one-to-one correspondence between templates and fragments, *i* is used as an index for both).
2. Construct the upper and lower inter-atomic distance bounds from the connection table.
3. Assign a weight *w_i_* to each atom *i*.

**Figure 1:**
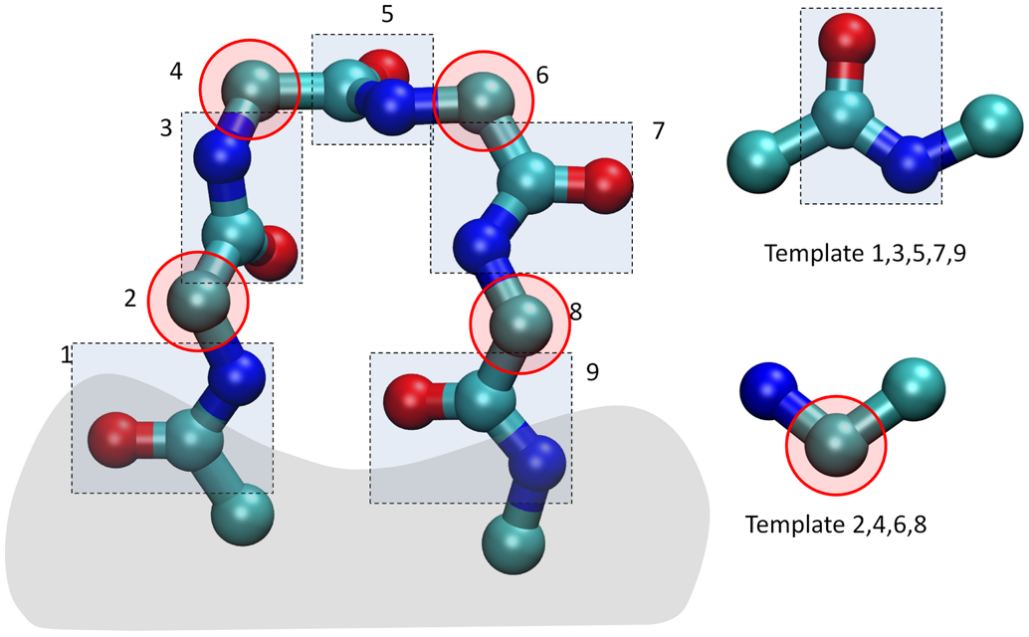
Decomposition of a 4-residue loop into a set of rigid fragments. The green, blue and red balls represent the carbon, nitrogen, and oxygen atoms, respectively. The gray area corresponds to the fixed part of the protein where the loop is anchored. The protein loop backbone can be decomposed into a series of alternating amide (in blue rectangular boxes) and methylene groups (in red elliptical boxes). The two structures on the right hand side are the corresponding reference templates with their attached atoms.

In order to identify conformationally rigid fragments, the program must first identify all rotatable bonds present in the molecule. For a general molecule, single acyclic bonds are assumed to be freely rotatable, unless they are part of a small ring (size 6 or smaller), or a delocalized system, such as the N-C bond in an amide group. The rotatable bonds are then removed, and the remaining sub-graphs (connected components) represent the rigid fragments. Figure 1 illustrates the fragments derived from the backbone of a short protein loop with four residues connected to a fixed part of the protein (area in shadow). The peptide backbone can be decomposed into an alternating series of amide (-NH-CO-) and C_α_ groups, each of which can be considered rigid. The conformations of these groups can be either extracted from a 3D database or determined from simple geometric constraints using SPE or other methods. For example, the geometry of the amide group can be uniquely determined by the bond lengths, bond angles, and planarity of the amide bond.

As in the original SOS algorithm, the fragment templates that serve as reference structures for the superimposition operations also include the atoms directly attached to them through rotatable bonds. This ensures that the resulting conformation preserves the correct relative orientation between fragments (Figure 1). However, while the coordinates of the core atoms in a fragment can be taken directly from the pre-computed templates, the coordinates of the attached atoms need further adjustment because in the reference templates they are represented by explicit hydrogens. This is achieved by replacing the corresponding hydrogens with the actual atoms in the molecule and adjusting the bond lengths accordingly. In our current implementation, the fixed part of the protein is treated as a single fixed fragment, translating the loop closure problem into a conformation generation problem for a cyclic molecule.

The calculation of the upper and lower interatomic distance bounds follows the standard procedure outlined in the original SPE and SOS algorithms [53],[59]. For atoms that are bonded to each other (1,2), bonded to a common third atom (1,3), or bonded to two atoms that are directly bonded themselves (1,4), the lower bound 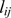 (where *i* and *j* are the indices of the atoms in question) is determined based on standard covalent geometry, otherwise it is set to the sum of their Van der Waals radii. The upper bounds 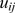 are usually set to the sum of the bond lengths along the shortest path connecting atoms *i* and *j*, obtained from the Floyd-Warshall algorithm.

#### Embedding

Once constructed, the templates are used in an iterative embedding procedure that involves successive template fits followed by pairwise adjustments of atomic positions to gradually refine the conformation of the molecule. The algorithm proceeds as follows:

1. Position the terminal atoms of the loop at their predefined distance. Place the remaining atoms at random positions in the vicinity of the terminal atoms.
2. **Repeat** *n_c_* times {
   3. **For each** fragment *F_k_* in the molecule **do** {
**   Repeat** *n_p_* times**   {**
      4. Reset the terminal atoms of the loop to their fixed positions.
     5. Pick a random pair of atoms *i* and *j* from two different fragments.
      Calculate the distance between atoms *i* and *j*, 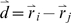, 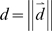.
     6. Retrieve the corresponding upper and lower distance bounds, *u_ij_* and *l_ij_*, between atoms *i* and *j*.
     7. Update the coordinates of atoms *i* and *j* as follows. If (*d*<*l_ij_*){
        Set 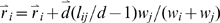.
        Set 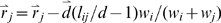.     }
      Else if (*d*>*u_ij_*)
      {
       Set 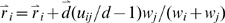.
       Set 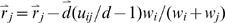.    }
   }
     8. Superimpose the template *T_k_* onto the existing conformation of fragment *F_k_*. Replace the coordinates of the atoms in *F_k_* with the corresponding coordinates in the superimposed template *T_k_*. Record the maximum distance deviation 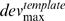.}
     9. Record the maximum end-point distance deviation, 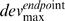. If 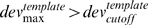 or 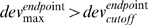, where 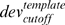 and 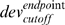 are prescribed thresholds, repeat step 3.}

**Figure 2:**
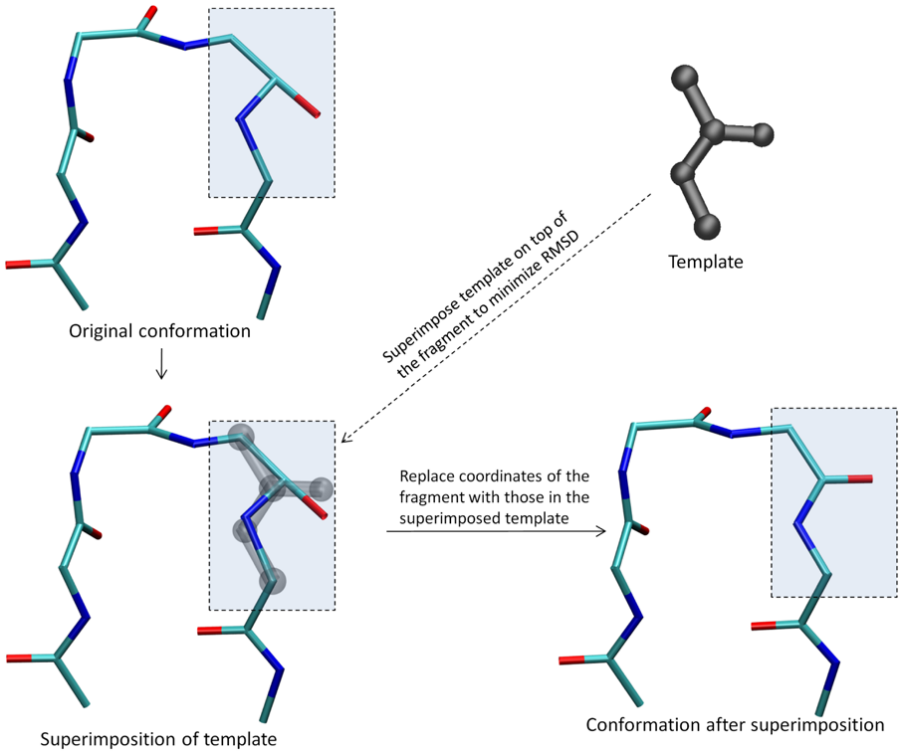
Schematic illustration of superimposition operation for an amide group in a 4-residue loop. First, one of the fragments is picked at random (shown in the rectangular box). Second, a weighted rigid-body alignment is performed to superimpose the template on top of the selected fragment. Finally, the coordinates of the fragment are replaced with those of the superimposed template, therefore ensuring the correct bond lengths, bond angles, and planarity for this fragment.

After assigning random initial coordinates to all the atoms in the loop, the SOS cycles begin by resetting the positions of the terminal atoms to the predefined fixed points to satisfy the anchor constraints. Every cycle iterates over all rigid fragments in random order and updates the coordinates of their constituent atoms by least-squares fitting of the corresponding template. Within each cycle, steric clashes are gradually removed by *n_p_* pairwise distance adjustments between any two successive fitting operations. Each pairwise adjustment selects a random pair of points and checks if their distance *d_ij_* falls within the prescribed lower and upper bounds, *l_ij_* and *u_ij_*. If not, the atoms are moved along their axis towards satisfying that constraint (i.e., towards each other if their current distance is larger than the upper bound, and away from each other if it is smaller than the lower bound). If the atoms are already within their prescribed bounds, their positions remain unchanged. The magnitude of the adjustment is inversely proportional to the atoms' weights, *w_i_* and *w_j_*. Properly assigned weights can promote the satisfaction of the fixed point constraints and accelerate convergence [59]. In effect, the superimposition operations correct the geometry of locally rigid substructures in a single conceptual step, while maintaining the proper chirality (Figure 2). Compared with the original SOS implementation, the current variant incorporates the anchor constraints for the terminal atoms of the loop. Moreover, rather than stopping after a predefined number of cycles, a more adaptive convergence criterion is applied. The maximum displacement for all the atoms in the template is recorded, and used to assess convergence. If the maximum atomic displacement across all templates and the end point distance violations are smaller than the prescribed thresholds 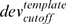 and 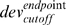 respectively, the innermost loop is considered successful. Because the pairwise adjustments are interlaced with the superimposition operations, it is possible that the locally optimal geometry obtained from the last fitting step is distorted by the subsequent pairwise adjustments and superimposition operations. Therefore, we consider the cycle successful after the completion of 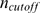 successive successful loops.

#### Weighted template superimpositions

The correct geometry of each fragment is enforced by repeatedly superimposing the corresponding template onto the fragment's current 3D configuration, and then copying the coordinates of the atoms in the superimposed template back to the original molecule. As we mentioned earlier, when two neighboring fragments are connected by a rotatable bond, that bond is included in both of them. Therefore, a superimposition operation of one fragment may distort the locally optimal geometry that resulted from a previous superimposition of one of its adjacent fragments. In order to alleviate this wasteful oscillation and improve the convergence rate, we assign a higher weight *w_1_* to the atoms along the connecting rotatable bonds, as we did in our previous study [59]. For the protein loop problem in particular, we assign a separate weight *w_2_* (*w_2_*≫*w_1_*) to the fixed atoms in order to minimize the deviation of the loop terminals from the fixed end points. The weighted template superimposition is illustrated in Figure 2. For a chosen fragment, we first perform a weighted rigid-body alignment to superimpose the corresponding template on top of that fragment, and then replace the fragment coordinates with those of the superimposed template.

The rate-limiting step in the SOS algorithm is the superimposition of templates. Let ***A*** and ***B*** denote the coordinate matrixes of the template and target fragment structures, where each row corresponds to the position of the *i*-th atom in the respective structure, (*x_A,i_*, *y_A,i_*, *z_A,i_*) and (*x_B,i_*, *y_B,i_*, *z_B,i_*). The weighted inner product of ***A*** and ***B*** is given by
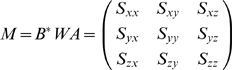
(1)where
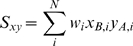
(2)and the matrix ***W*** is the diagonal matrix with each diagonal element being the weight 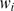 we discussed above.

Horn has showed that the quaternion of the optimal rotation matrix that minimizes the root mean square deviation between two structures A and B is the eigenvector associated with the largest positive eigenvalue of the symmetric matrix Q [60]:
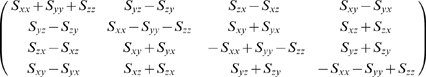
(3)


Instead of solving this eigensystem with the traditional Householder reduction method followed by QL decomposition with implicit shift [61] as used in the original SOS algorithm [59], we adopt the Newton-Raphson quaternion-based characteristic polynomial algorithm, an approach developed by Theobald and reported to be orders of magnitude faster than the traditional eigen decomposition approach [62]. Essentially, we first solve the characteristic polynomial with the Newton-Raphson algorithm for the largest eigenvalue, and then use cofactor matrices to calculate the corresponding eigenvector, which can easily be converted into the optimal rotational matrix needed for the superimposition. This new approach of determining the rotation matrix results in a 100% speedup compared to the original SOS algorithm.

#### Computational details and test set

The algorithm was implemented in C++ and is part of the DirectedDiversity software suite [55], which is in turn part of the Third Dimension Explorer and ABCD informatics offering [63]. To validate our algorithm, we compared it with the CCD method recently developed by Canutescu and Dunbrack [40] and the CSJD method by Coutsias *et al.* [41]. To simplify comparison, we used the same data set that was employed in both works, which consists of three sets of loops of length 4, 8, and 12, containing 10 different loops each. All SOS conformations were generated using the following parameters: *n_p_* = 3 (number of pairwise distance adjustments between two successive superimposition operations); *w_1_* = 5 (weights assigned to the atoms in the rotatable bonds); *w_2_* = 500 (weights assigned to the fixed terminal atoms); 
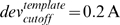
 (maximum allowed displacement during the fitting operation); 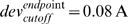 (maximum allowed fixed end-point deviation); and 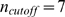 (number of successive successful cycles for SOS to be considered converged). These parameters were chosen based on the following considerations. The parameter n_p_ was tested on the 1cruA_358 12-residue loop to ensure sufficient distortion before the template superimposition, and was then used for all the remaining loops. The weight *w_1_* was directly adopted from the original SOS paper since there is no fundamental difference between the fragments from small organic molecules and peptides. The weight *w_2_* was set to an arbitrary large number, which essentially made the anchor points immobile. (An alternative weight of 1000 was also tested, but no substantial difference was observed.) The parameter 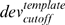 was chosen so as to prevent substantial structural distortion but still allow some flexibility. Three values, 0.05, 0.2, and 0.4 Å, were tested on the 1cruA_358 12-residue loop, and the value of 0.2 Å was found to be the most appropriate and applied to all the remaining loops. The parameter 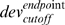 was adopted directly from the original cyclic coordinate descent paper. Finally, the parameter 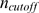 was chosen so as to produce an ensemble of physically plausible conformations. The smaller the value, the faster the convergence and the higher the probability for the conformation to be distorted. Several values were tested on the 1cruA_358 12-residue loop (1, 3, 5, and 7), and the value of 7 was found to be a reasonably conservative choice.

All calculations were performed on an IBM Thinkpad T61 laptop computer equipped with a single dual-core 2 GHz mobile Intel processor and 1.96 GB 667 MHz DRAM (using only a single core).

## Results

**Figure 3:**
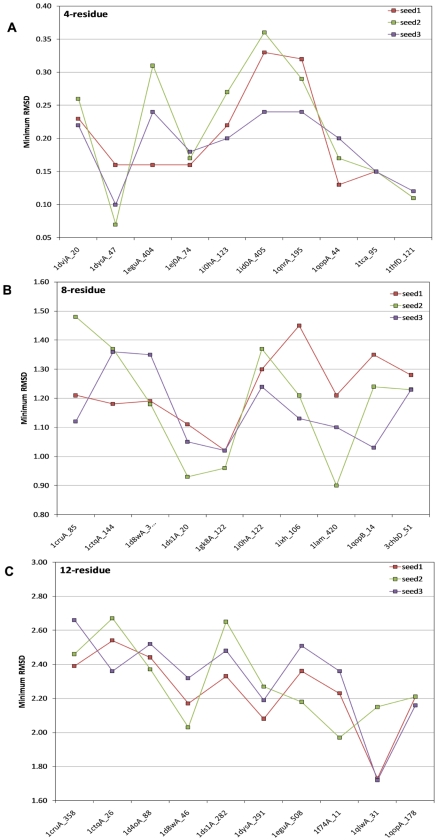
Minimum RMSD of the 5,000 conformers generated for each loop from their respective X-ray structures. The three series represent three independent SOS runs, each starting from a different random number seed and resulting in a different set of 5,000 conformers. The results are presented in 3 different panels for clarity. (A) 4-residue loops; (B) 8-residue loops; (C) 12-residue loops.

To allow a direct comparison with the CSJD and CCD algorithms, 5,000 different conformations were generated for each of the 30 representative loops, and the RMSD of each of these conformations to the known crystal structure was recorded. To further demonstrate the robustness of our algorithm, three different sets of simulations (i.e., three sets of 5,000 conformations) were performed for each loop, each starting from a different random number seed. The minimum RMSD's to the X-ray structures among the 5,000 conformations associated with each run (where a run denotes the 5,000 conformations generated from a particular random seed) are illustrated in Figure 3. The plot is divided into three panels for the 4, 8 and 12-residue loops respectively. The loop labels are composed of the PDB name of the proteins in which they were found, followed by their starting positions in the amino acid sequence. The y axis shows the best RMSD values calculated for the backbone atoms N, C, O and C_α_. The three lines in each panel represent the results for each independent run (random seed). As seen from these plots, the observed variability is relatively small and within the limits expected from the stochastic nature of the method. From these three independent simulations, the current algorithm produced consistently good backbone conformations with a mean best RMSD of 0.20, 1.19, and 2.29 Å, and the average sample size required to produce the best RMSD conformation was 2493, 2316, and 2761 for short (4-residue), medium (8-residue), and long (12-residue) loops, respectively.

To enable a more direct comparison, the results from the first run are listed in Table 1, along with the values obtained by the CSJD and CCD methods. The average minimum RMSD's across all 10 loops of a given size were 0.20, 1.19, and 2.25 Å for the 4, 8, and 12-residue loops, respectively, compared to 0.56, 1.59, and 3.05 Å for CCD, and 0.40, 1.01, and 2.34 Å for CSJD. Although 5,000 conformations were generated for each loop, in the majority of cases the best structure was identified within the first 3,000 SOS trials. As seen in Figure 3, these values can be further improved if another random seed is employed. We have previously shown that SPE and SOS are considerably more effective than other methods in sampling the full range of conformations available to a given molecule [53],[59], and it is to be expected that there will be less variability as the conformational space gets saturated (the number of unique conformations levels off asymptotically as the number of trials increases).

**Table 1:** Minimum RMS from X-ray structures for three different algorithms.

4-residue Loops				8-residue Loops				12-residue Loops			
Loop	SOS	CSJD	CCD	Loop	SOS	CSJD	CCD	Loop	SOS	CSJD	CCD
1dvjA_20	0.23	0.38	0.61	1cruA_85	1.48	0.99	1.75	1cruA_358	2.39	2.00	2.54
1dysA_47	0.16	0.37	0.68	1ctqA_144	1.37	0.96	1.34	1ctqA_26	2.54	1.86	2.49
1eguA_404	0.16	0.36	0.68	1d8wA_334	1.18	0.37	1.51	1d4oA_88	2.44	1.60	2.33
1ej0A_74	0.16	0.21	0.34	1ds1A_20	0.93	1.30	1.58	1d8wA_46	2.17	2.94	4.83
1i0hA_123	0.22	0.26	0.62	1gk8A_122	0.96	1.29	1.68	1ds1A_282	2.33	3.10	3.04
1id0A_405	0.33	0.72	0.67	1i0hA_122	1.37	0.36	1.35	1dysA_291	2.08	3.04	2.48
1qnrA_195	0.32	0.39	0.49	1ixh_106	1.21	2.36	1.61	1eguA_508	2.36	2.82	2.14
1qopA_44	0.13	0.61	0.63	1lam_420	0.90	0.83	1.60	1f74A_11	2.23	1.53	2.72
1tca_95	0.15	0.28	0.39	1qopB_14	1.24	0.69	1.85	1qlwA_31	1.73	2.32	3.38
1thfD_121	0.11	0.36	0.50	3chbD_51	1.23	0.96	1.66	1qopA_178	2.21	2.18	4.57
Average	0.20	0.40	0.56	Average	1.19	1.01	1.59	Average	2.25	2.34	3.05

CSJD and CCD results were obtained from Table 1 and Table 2 of ref [41] and ref [40], respectively. As in CCD, 5,000 trials were performed for each test loop in our SOS calculations. However, the majority of minimum RMSD's were reached within the first 3,000 trials. All the results reported here came from a single run per loop, using the same random seed. Some of these values can be improved if a different seed is chosen.

It is also worth noting that the results obtained with our algorithm are more consistent than those obtained by CSJD and CCD. For instance, for all ten of the 12-residue loops, the minimum RMSD's obtained by SOS were always less than 2.55 Å, whereas for the CSJD and CCD algorithms these values ranged as high as 3.10 and 4.83 Å, respectively. Because in the realistic loop prediction problem there is no reference structure to compare against, this observation gives us more confidence that the actual loop structure will be close to at least one of the structures identified by our algorithm. Clearly, the larger the loop, the greater its conformational flexibility, and the greater the number of trial conformations one needs to generate in order to adequately sample the space.

**Figure 4:**
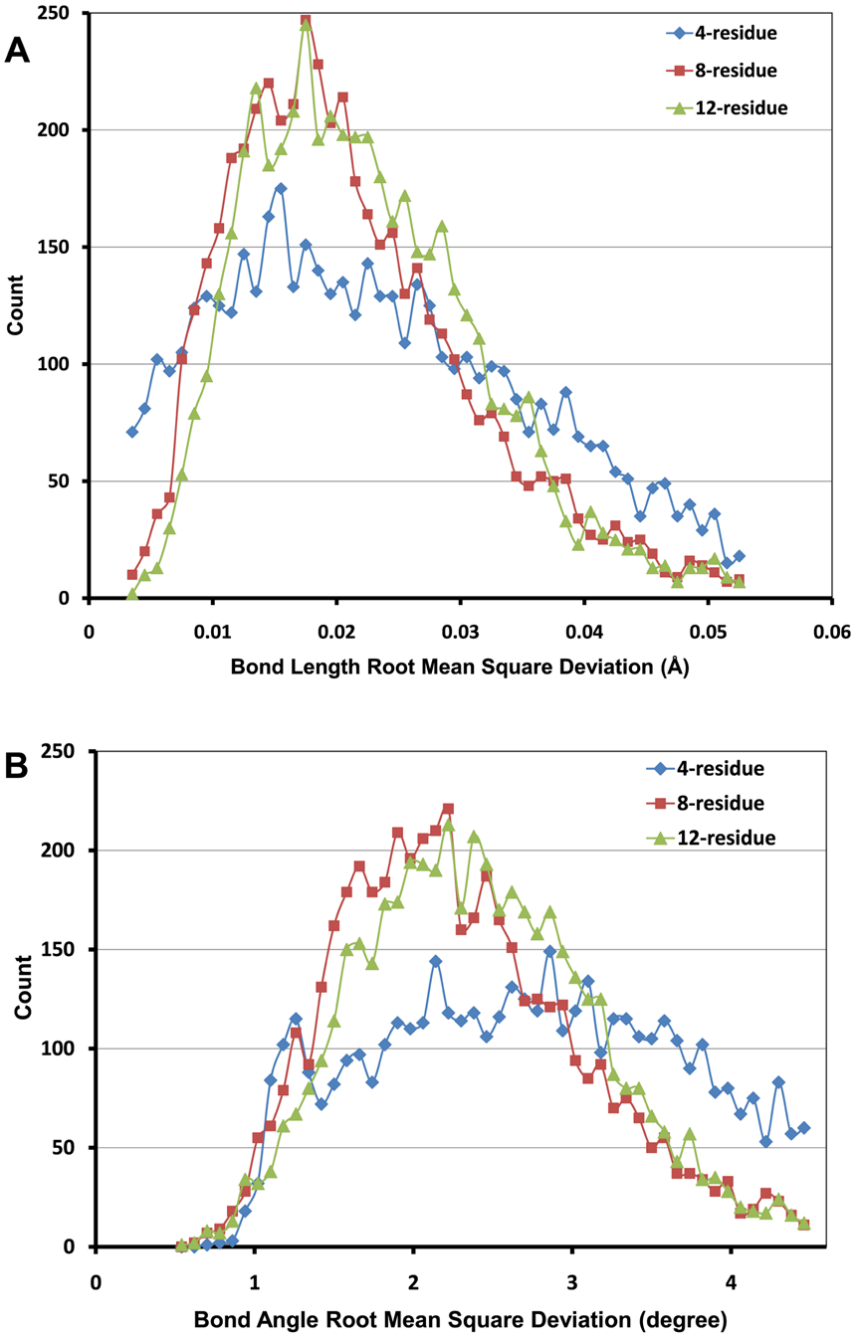
Histogram of the root mean square deviations of bond lengths and angles. The histograms are generated from all conformations for a given loop size. (A) Bond lengths, and (B) bond angles.

To assess the quality of the entire conformational ensemble generated by the SOS algorithm, the root mean square deviations of all bond lengths and angles in the resulting loop conformations were calculated against their ideal values, and the resulting distributions were plotted in Figure 4 (bond lengths in the top panel, bond angles in the bottom). The three series in each panel represent the combined distributions of the 4, 8, and 12-residue loops, respectively. As is evident from these distributions, the bond lengths were reproduced remarkably well, with the majority of the deviations limited to less than 0.02 Å and the overwhelming majority less than 0.04 Å. This is a very satisfactory result, considering that the σ bonds between two carbon atoms can vary from 1.49 Å to 1.54 Å [59] and that an even larger variation is observed in the crystal structures deposited in the Protein Data Bank. Similarly, the majority of conformations show very small bond angle deviations (less than 3 degrees). Interestingly, the distribution of angle deviations is slightly broader for the 4-residue loops, which probably reflects their more constrained nature and the relatively greater difficulty in meeting the end point constraints.

**Figure 5:**
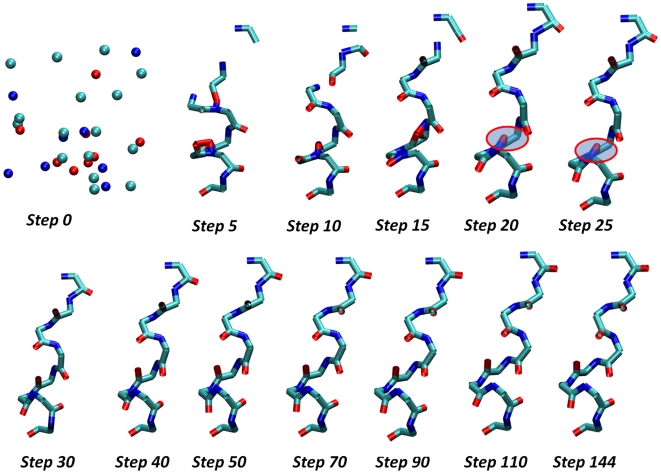
Representative snapshots of the conformation generation process for an 8-residue loop. Starting from a completely random conformation (step 0), the SOS algorithm drives the atoms in the vicinity of their ideal locations within only 5 steps. At the end of step 20, the conformation has only one steric clash, as highlighted by the red ellipse. This clash is gradually resolved within 10 additional steps. The simulation continues until it converges at step 144. By relaxing our rather stringent convergence criteria, significant speedup can be achieved without a significant impact on the quality of the resulting conformation.

To illustrate how the molecular geometries are improved during the course of the SOS refinement, Figure 5 shows a few representative snapshots of a single 8-residue loop refinement run. Starting from a random initial conformation (Step 0), the SOS procedure rapidly drives the atoms close to their final locations within only 5 refinement steps. After 20 steps, the loop conformation is successfully constructed with only one steric clash. This clash is gradually resolved within a few more steps. The conformation is only slightly adjusted beyond Step 30 to satisfy the strict convergence criteria, which are fully satisfied in Step 144.

A practical and useful algorithm must strike a good balance between the quality of conformations that it generates and the computational time expended. The efficiency of the SOS algorithm was evaluated by calculating the average time required to generate 5,000 conformations for all ten protein loops in each set. The computing time per conformation averaged over 5,000 conformations for each 4, 8 and 12-residue loop was 4.5, 12, and 17 milliseconds, respectively, on a single 2 GHz mobile Intel processor (using only one of the two available cores). In addition to giving better average minimum RMSDs (0.20, 1.19, and 2.25 Å for SOS, 0.56, 1.59, and 3.05 Å for CCD, and 0.40, 1.01, and 2.34 Å for CSJD for the 4, 8, and 12-residue loops, respectively), the current approach is more efficient than CCD. Indeed, SOS required 5.0, 13, and 19 ms when scaled to the same processor (AMD 1800+ MP), compared to 31, 37, and 23 ms for CCD for the 4, 8 and 12-residue loops. Although the efficiency is not as impressive as the CSJD algorithm's (0.56, 0.68, and 0.72 ms on an AMD 1800+ MP processor), it is more than sufficient for virtually all practical uses. It is worth mentioning that the “numerical” closure, which is essentially the conformational sampling scheme used in PLOP [33], gave very good RMSD's (0.27, 1.04, and 1.89 Å) with an average computing time of 8.5, 6.1, and 23 ms per loop for the 4, 8, and 12-residue loops [41]. Since the SOS algorithm resolves steric clashes during the course of the refinement through the use of pairwise distance adjustments, the resulting conformations are chemically and geometrically “clean”, and ready for use in more detailed investigations. It is worth pointing out that the efficiency of our algorithm can be substantially improved by employing less stringent convergence criteria. As seen in Figure 5, if the simulation is stopped at Step 30, the efficiency will be enhanced by a factor 5 without a significant impact on the quality of the resulting geometries.

## Discussion

In this article, we introduced a conceptually simple, fast and robust solution to the well-known loop closure problem. By performing fast weighted superimpositions of rigid fragments and adjusting the distances between randomly chosen atoms to resolve steric clashes, this method can efficiently generate chemically sensible geometries that satisfy end point, steric, planar and chiral constraints. Once the templates are constructed, their correct chirality and planarity is naturally preserved through the template fitting operations.

Compared to other loop construction algorithms, the advantages of the current approach lie on its conceptual simplicity, computational efficiency, numerical stability and ease of implementation. Unlike alternative methods which generate new conformations by randomly perturbing the current structure, our algorithm always starts from completely random initial coordinates and there is no correlation whatsoever between successive conformations. Moreover, our method does not necessitate an existing three-dimensional conformation as input, but only the loop's sequence (connection table). More importantly, it is straightforward to incorporate additional distance constraints, making the approach especially suitable for protein structure determination using NMR and other methods. Non-covalent interactions such as hydrogen bonds can be encoded using additional distance constraints, making possible the detection of multiple interlocking rings in protein loop regions. This represents a tremendous challenge for conventional loop closure algorithms, but the SOS algorithm handles it naturally without any additional algorithmic modifications.

The only possible disadvantage of the SOS method is its reliance on pre-computed conformational templates. A method for extracting such templates from an existing set of molecules into a 3D fragment library has already been presented [59]. But for the protein loop closure problem the task is actually trivial, since the entire protein can be built from just a few rigid fragments, whose conformations can be either directly extracted from known protein structures or generated from other conformation sampling algorithms such as SOS and SPE.

The algorithm described here can be used to generate good quality conformations for protein loops of any length. Its efficiency makes it ideally suited for homology modeling where speed is critical. By relaxing the convergence criteria, the loop building process can be further accelerated without a significant worsening of the resulting conformations. Our approach could also be used as a means of generating local moves in a Markov Chain Monte Carlo simulation. The extension of this approach to include crystal contacts, side chains and other non-covalent interactions is currently under investigation.
